# Circadian Rhythm: Biological Functions, Diseases, and Therapeutic Targets

**DOI:** 10.1002/mco2.70435

**Published:** 2025-10-22

**Authors:** Kangkang Zha, Bobin Mi, Yuan Xiong, Shuyan Wu, Li Lu, Shengming Zhang, Xuan Lu, Hei Chung Mak, Jianping Huang, Adriana C. Panayi, Samuel Knoedler, Lili Chen, Guohui Liu, Sien Lin

**Affiliations:** ^1^ Department of Orthopaedics Union Hospital Tongji Medical College Huazhong University of Science and Technology Wuhan China; ^2^ Hubei Province Key Laboratory of Oral and Maxillofacial Development and Regeneration Wuhan China; ^3^ Department of Orthopedic Surgery Tongji Hospital Tongji Medical College Huazhong University of Science and Technology Wuhan China; ^4^ Department of Orthopaedics & Traumatology Stem Cells and Regenerative Medicine Laboratory Li Ka Shing Institute of Health Sciences The Chinese University of Hong Kong Hong Kong SAR China; ^5^ Department of Prosthodontics Yonsei University College of Dentistry Seoul South Korea; ^6^ Division of Plastic Surgery Brigham and Women's Hospital Harvard Medical School Boston Massachusetts USA; ^7^ Department of Stomatology Union Hospital Tongji Medical College Huazhong University of Science and Technology Wuhan China; ^8^ Department of Orthopaedics & Traumatology School of Clinical Medicine LKS Faculty of Medicine The University of Hong Kong Hong Kong China

**Keywords:** circadian rhythm, circadian disruption‐related diseases, clock gene, therapy, tissue homeostasis

## Abstract

Circadian rhythms are intrinsic 24‐h biological cycles that govern various physiological processes. In addition to the hypothalamic suprachiasmatic nucleus, the circadian system is organized in multiple peripheral tissues, such as the brain, heart, bone, liver, and lung. Emerging evidence suggests that disruptions in these rhythms, which are regulated by a network of clock genes, play pivotal roles in human health. A deeper understanding of the interplay between circadian rhythms and tissue homeostasis holds significant potential for the development of targeted therapies aimed at improving human health. This review explores the link between circadian rhythms and tissue homeostasis, delving into their biological functions, including influences on metabolic homeostasis, neuroendocrine signaling, immune and oxidative stress responses, tissue repair, and autophagy activity. It also summarizes the connections between circadian disruptions and circadian disruption‐related diseases, including degenerative diseases, cardiometabolic disorders, and cancers. Furthermore, this review offers valuable perspectives on the treatment of circadian disruption‐related diseases. By revealing the regulatory influence of circadian rhythms on human health and disease, this work aims to inspire the development of novel strategies for the prevention, diagnosis, and treatment of circadian disruption‐related diseases.

## Graphical abstract

This review offers a comprehensive overview of the mechanisms by which circadian rhythms modulate tissue homeostasis, alongside the associations between circadian disturbances and circadian rhythm‐related diseases. Furthermore, we offer insights into circadian rhythm‐regulating therapies, which may contribute to the development of novel strategies for the prevention and treatment of circadian rhythm‐related diseases.

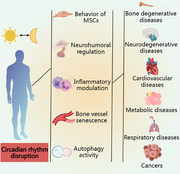

## 1 Introduction

The circadian rhythm is the endogenous timing system prevalent in various species and regulates cascades of physiological and metabolic processes [1, 2]. Many hypothalamic nuclei are circadian rhythm regulators, although the circadian rhythm is largely controlled by a self‐sustained circadian pacemaker located in the hypothalamic suprachiasmatic nucleus (SCN) [3]. The hypothalamic paraventricular nucleus, which harbors neurons that control the activity of the sympathetic and parasympathetic nervous systems, is a key target of biological clock output [4]. Furthermore, the secretion of hormones, such as cortisol and melatonin, is also regulated by the internal biological clock, which is entrained by the SCN [5]. The circadian rhythm within the SCN could affect peripheral tissues via both the neuronal and the endocrine pathways [6, 7, 8]. Recent findings have shown that the circadian system is organized in multiple peripheral tissues, such as the brain [9], heart [10], liver [11], lungs [12], skin [13], and skeletal muscle [14], in addition to the SCN. The circadian oscillators in peripheral tissues can be temporarily lost because of significant and abrupt environmental alterations [15].

Long‐term circadian disruptions can lead to multiple disorders. For example, Gil‐Lozano et al. [16] evaluated the association between intestinal L‐cell function and circadian rhythms and reported that sleep deprivation with nocturnal light exposure induced increased insulin resistance and disrupted melatonin and cortisol profiles in male volunteers. Wilms et al. [17] demonstrated that significant changes occurred in human white adipose tissue following circadian disruption due to sleep deprivation. These changes included the loss of rhythmicity in carbohydrate breakdown‐related genes, decreased secretion by β‐cells, and increased expression of retinol‐binding protein 4 [17]. A natural circadian rhythm is highly important for maintaining normal physiology and metabolism.

In mammalian cells, the circadian rhythm is controlled by a core group of regulatory genes, namely, clock genes, whose expression has been characterized mainly on the basis of circadian patterns [18, 19]. The underlying mechanisms through which circadian rhythm disruption causes diseases have been revealed in part and are considered to be associated with disturbances in proteostasis, oxidative stress, and immune function [9]. A deeper understanding of the interplay between circadian rhythms and tissue homeostasis holds significant potential for the development of targeted therapies aimed at combating circadian disruption‐related diseases (CDDs).

In this review, we first introduce the relationship between circadian rhythm and tissue homeostasis. Next, we focused on the associations between circadian disturbances and CDDs, as well as the therapeutic targets aimed at addressing circadian rhythm disturbances and CDDs. Finally, we provide insights that can be gained regarding the role of the circadian rhythm in tissue homeostasis, which will be conducive to the treatment of CDDs.

## 2 Molecular Architecture of the Circadian Clock

### 2.1 Core Clock Genes/Proteins and Transcription‒Translation Feedback Loops

**FIGURE 1:**
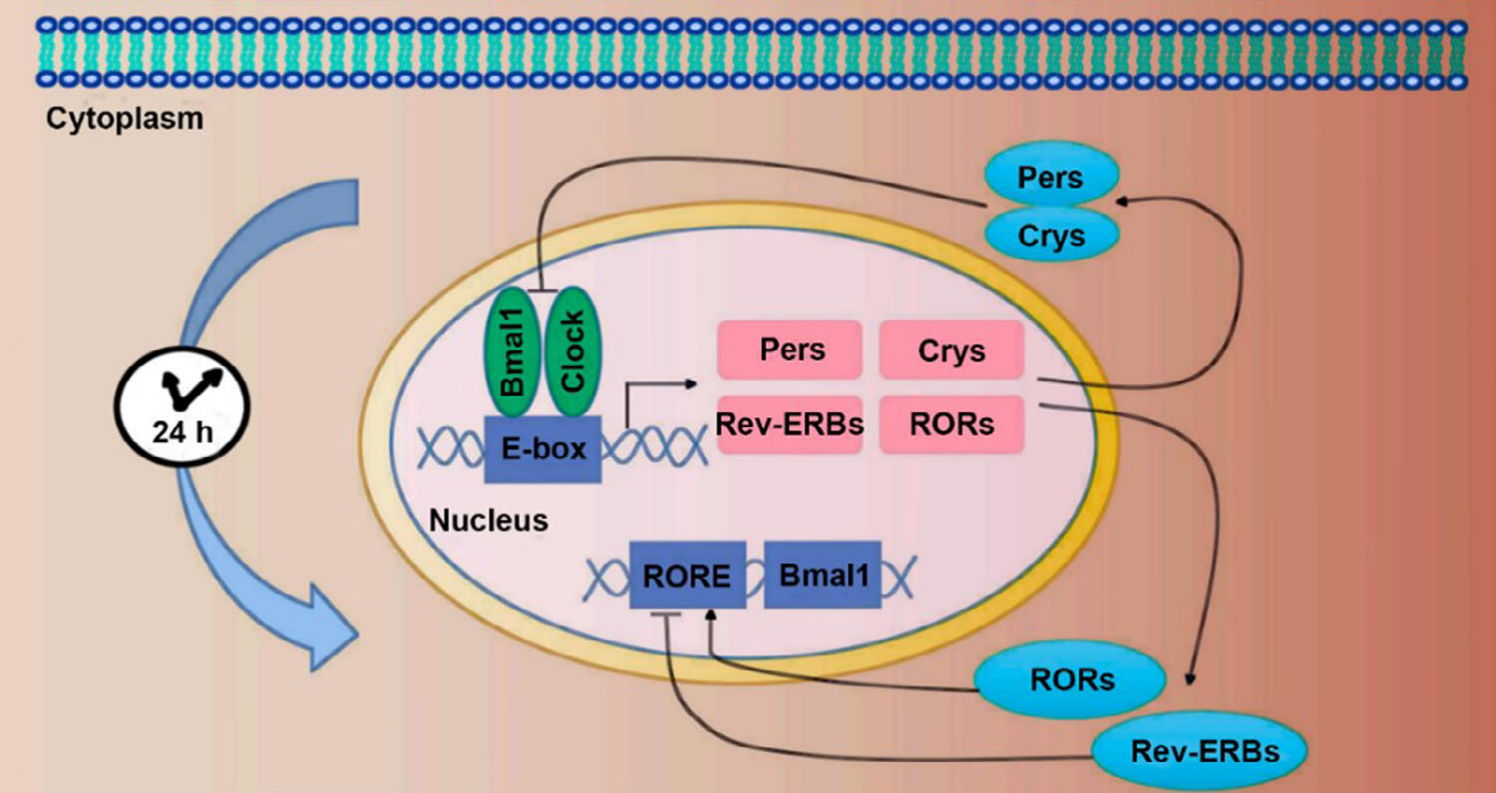
Two mammalian core clock gene feedback loops: Bmal1/Clock–Per/Cry and Bmal1–Rev‐ERB/ROR. Bmal1, brain and muscle ARNT‐like protein‐1; Clock, circadian locomotor output cycles; Cry, cryptochrome; Per, period circadian clock; ROR, retinoid‐related orphan receptor; RORE, ROR response element.

In mammalian cells, the circadian rhythm is controlled by a core group of clock genes, including brain and muscle ARNT‐like protein‐1 (Bmal1), circadian locomotor output cycles (Clock), Period (Per), Cryptochrome (Cry), REV‐ERB, and retinoid orphan nuclear receptor (ROR) [18, 19]. Various rhythmic genes and their encoded products form feedback loops involving mutual promotion or inhibition of transcriptional and translational activities to ultimately achieve periodic rhythmicity at the cellular and molecular levels (Figure 1). Transcription‒translation feedback loops serve as the core driving force behind the molecular circadian clock. This evolutionarily conserved mechanism is primarily composed of two interlocking feedback loops that act synergistically to ensure the precision, periodicity, and stability of circadian oscillations.

The core negative feedback loop establishes the fundamental framework of the system. Within this loop, the core transcription factors Bmal1 and Clock form a functional heterodimer (Bmal1:Clock) inside the nucleus. This complex, which acts as the primary transcriptional activator, efficiently initiates the transcription of various clock‐controlled genes, including the Per and Cry genes, by binding to E‐box elements in their promoter regions [20, 21]. Following translation, the resulting Per and Cry proteins must first undergo a series of phosphorylation events that ensure their stability, after which they accumulate in the cytoplasm, form a heteromeric complex, and are transported back into the nucleus. Once inside, the Per:Cry complex directly inhibits the transcriptional activity of Bmal1:Clock, thereby forming a classic negative feedback loop that effectively “switches off” their own transcription and clears the way for a new transcriptional cycle to begin [22].

Concurrently, a parallel auxiliary loop provides more sophisticated dynamic regulation to the core loop, enhancing its stability and robustness. This loop centers on the rhythmic expression of the Bmal1 gene itself. The transcription factors REV‐ERB and the ROR competitively bind to the ROR response element (RORE) on the Bmal1 gene promoter. A direct antagonistic relationship exists between the two proteins: REV‐ERB binding to the RORE represses Bmal1 transcription, whereas ROR binding promotes it [23, 24].

In short, Bmal1 and Clock form a heterodimer that localizes to the nucleus and can activate the transcription of the Per and Cry genes; the products of these two genes negatively regulate the transcription of the progenitor heterodimer. In addition, the Bmal1, REV‐ERB, and ROR genes constitute a regulatory feedback loop, and Bmal1 transcription is negatively regulated by REV‐ERB and positively regulated by ROR. The synergistic action of these two loops ultimately results in a highly robust cellular circadian oscillator.

### 2.2 Posttranslational Modifications

Posttranslational modifications are key molecular mechanisms linking the biological clock to downstream physiological and pathological processes. Among them, phosphorylation and ubiquitination play pivotal roles in rhythm‐mediated metabolic homeostasis.

As a rapid and reversible posttranslational modification, phosphorylation functions as a “molecular switch,” modulating protein activity by altering protein conformation. This mechanism precisely calibrates the circadian clock's regulation of metabolism and cellular behavior. Phosphorylation directly influences the function of core clock proteins. The phosphorylation of Bmal1 is essential for its functional diversity. For example, phosphorylation at the serine 42 residue allows Bmal1 to exert its effects at synapses outside the cell nucleus, thereby impacting synaptic plasticity [25]. In the context of metabolism, a decrease in the phosphorylation level of Bmal1 is negatively correlated with various adverse metabolic and inflammatory markers, indicating a strong association with the pathophysiology of type 2 diabetes [26].

Ubiquitination plays a multifaceted role within the circadian system, not only regulating the core clock loop but also mediating its downstream physiological outputs. In the context of the core circadian feedback loop, the Bmal1:Clock complex marks the downstream Per1 and Per2 genes through histone monoubiquitination, a fundamental mechanism that facilitates negative feedback regulation of circadian rhythms [21]. Under physiological conditions, the biological clock maintains tissue homeostasis by regulating ubiquitination. For example, in muscle tissue, the clock drives the rhythmic expression of the E3 ubiquitin ligase MuRF genes, selectively enhancing ubiquitination activity during the night to limit excessive muscle growth and promote long‐term muscle health [27].

### 2.3 Systemic Synchronizers

The SCN of the hypothalamus serves as the body's primary circadian pacemaker, integrating external light cues to generate an endogenous, self‐sustaining 24‐h rhythm [3]. The SCN communicates its rhythmic signals to the entire body through two major pathways, neural and endocrine pathways, thereby synchronizing the biological clocks in peripheral tissues and organs [6, 7, 8]. It governs the rhythmic secretion of several key hormones, such as cortisol and melatonin, establishing itself as a critical hub linking central rhythms with peripheral metabolism [28, 29, 30]. Recent studies have further emphasized the importance of the robustness of the SCN, revealing that the mitochondrial health of its neurons, as well as the expression levels of specific neuropeptides such as vasoactive intestinal peptide, is essential for maintaining systemic rhythmic homeostasis and the overall health of the nervous system [31, 32].

Various hormones exhibit distinct diurnal rhythms and serve as crucial signaling molecules regulating systemic metabolism [33, 34, 35]. For example, the secretion rhythm of cortisol peaks in the early morning to awaken the body and falls to a nadir at night [36]. Rhythmic disruption can directly disturb hormonal balance, subsequently triggering a series of pathological changes. For example, chronic circadian disturbances, such as social jetlag, can disrupt the normal secretion pattern of prolactin, promoting pathological lipogenesis in the liver and leading to hepatic steatosis [37]. Similarly, sleep deprivation can impair the balance between the appetite‐suppressing hormone leptin and the hunger‐stimulating hormone ghrelin, causing the brain to receive a false “energy deficit” signal, thereby intensifying hunger and cravings and ultimately leading to weight gain [38].

The biological functions of the circadian rhythm in peripheral tissues can be reflected through changes in metabolites. For example, bone turnover markers (BTMs) are metabolites formed during bone synthesis and catabolism. They reflect the processes of bone formation and resorption and can be used not only to diagnose metabolic bone disease and evaluate therapeutic effects but also to predict the rates of bone loss and fracture risk [39, 40]. Almost all existing BTMs exhibit diurnal and nocturnal rhythms [41, 42]. Biological rhythms integrate environmental signals, such as light and food intake, with endogenous metabolic demands. Metabolites serve not only as products of biological clock regulation but also as sensors for feedback regulation. Disruption of this dynamic balance is a significant contributor to metabolic diseases.

## 3 Physiological Functions Regulated by the Circadian Rhythm

**FIGURE 2:**
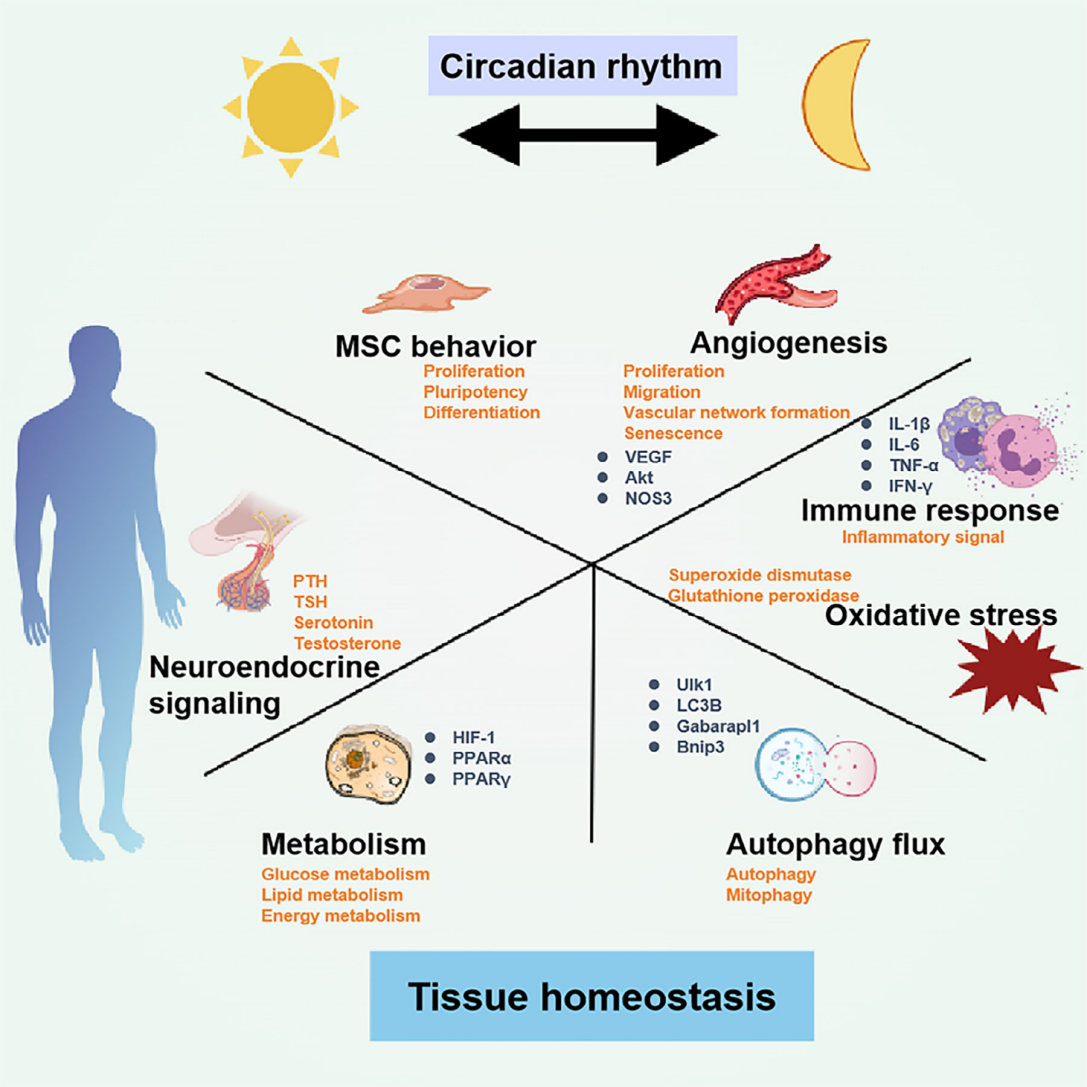
The biological function of the circadian rhythm. The circadian rhythm is involved in the regulation of different functions, including metabolism, neuroendocrine signaling, immune and oxidative stress responses, tissue repair, and autophagy activity, thus impacting tissue homeostasis.

In recent decades, a growing body of research has confirmed that circadian rhythms significantly influence tissue homeostasis. The effects of circadian rhythms on tissue homeostasis may be mediated through the modulation of metabolic homeostasis, neuroendocrine signaling, immune responses, oxidative stress, tissue repair mechanisms, and autophagy activity (Figure 2). These aspects are discussed in detail in the following sections.

### 3.1 Metabolic Homeostasis

The circadian rhythm is acknowledged as a vital regulator that preserves metabolic homeostasis, which is essential for the metabolism of glucose, lipids, and energy [43]. Compared with healthy individuals, those with established insulin resistance or type 2 diabetes might be more susceptible to the detrimental impacts of circadian disturbances on glucose metabolism [44, 45]. The liver and skeletal muscle are two critical organs for glucose metabolism, and both are regulated by the circadian rhythm. The interplay between liver clocks and skeletal muscle clocks plays an important role in supporting systemic glucose tolerance [46]. Interestingly, it has been demonstrated that the liver clock drives glucose clearance and gluconeogenesis via signals from gut microbes [47]. On the other hand, Bmal1 modulates skeletal muscle glycolysis and metabolic flexibility by regulating the HIF‐1 signaling pathway. Its specific knockout leads to dysregulation of glucose and the development of high‐fat‐diet‐induced glucose intolerance [48].

The results of metabolomics and lipidomics analysis revealed that numerous lipid species in human plasma, including fatty acids, triacylglycerols, glycerophospholipids, sterol lipids, and sphingolipids, are regulated by circadian rhythms [49]. The circadian clock is crucial for controlling the activities of PPAR proteins in the liver. Notably, PPARα enhances mitochondrial fatty acid β‐oxidation by interacting with the transcriptional coactivator PPARγ coactivator 1α (PGC‐1α) [50, 51]. Moreover, the Bmal1:Clock transcriptional complex increases the expression of genes encoding adipose triglyceride (TG) lipase and hormone‐sensitive lipase, thereby regulating the lipolysis rate throughout the circadian cycle [52]. In white adipose tissue, the nuclear receptor PPARγ is subject to circadian regulation, which significantly contributes to fatty acid synthesis and storage processes [53]. Disruption of the circadian clock encourages the buildup of TGs in white adipose tissue, resulting in greater adiposity and hypertrophy of adipocytes [54]. Skeletal muscle in healthy individuals also displays a rhythmic pattern in lipid metabolism that corresponds to the day‒night cycle [55]. Circadian disruptions influence the skeletal muscle lipidome, particularly by affecting triacylglycerols, especially those with a carbon length of 55 or more. These findings underscore the critical role that the circadian rhythm plays in the lipid metabolism of skeletal muscle [56]. More research should be conducted to further analyze the specific molecular interfaces of metabolite–rhythm interactions to identify new therapeutic targets for the treatment of metabolic diseases.

### 3.2 Neuroendocrine Signaling

Emerging evidence suggests that the circadian rhythm regulates the secretion of neurohormones. Thyroid hormones are known to be essential for organ development and growth [57]. A circadian rhythm in serum Parathyroid hormone (PTH) levels has been demonstrated in healthy people [35]. Disruption of the PTH circadian rhythm has been linked to the development of CDDs [58, 59, 60]. The relationship between Thyroid stimulating hormone (TSH) levels and circadian rhythm was investigated by Coppeta and coworkers [61], who reported that TSH was significantly greater in night workers than in day workers. Previous studies have demonstrated that the 24‐h mean plasma TSH level in older men is approximately 50% lower than that in young men [62]. It has also been revealed that the diurnal rhythm of TSH is altered in older rats compared with young rats [63]. These alterations reflect a certain role of aging‐related TSH circadian disruption in regulating human health and tissue degeneration.

Serotonin, also known as 5‐OH tryptamine, is produced from tryptamine in the brain, gut, and bone. There is evidence that the level of serotonin in the brain exhibits circadian rhythmicity [64]. Moreover, Jagota and Kalyani [65] demonstrated that daily secretion rhythmicity in the brain began at 3 months in rats but disintegrated at 6 months, indicating that age induced changes in the serotonin circadian rhythm. Compared with that in normal rats, the secretion of serotonin in rats with a disrupted circadian rhythm has been demonstrated to be decreased [66]. In a comparative study, Razavi et al. [67] reported that rotating‐shift workers exposed to greater light during night shifts presented lower urinary melatonin levels at night than did day‐shift workers did, indicating that melatonin secretion is influenced by the circadian rhythm. The circadian rhythm alterations in melatonin concentration during aging have been studied. Compared with young subjects, middle‐aged subjects presented decreased melatonin peak levels but longer peak levels [68].

Testosterone is another critical hormone that impacts human health [69, 70]. The effect of circadian rhythm disruption on testosterone synthesis has been investigated. Liu et al. [71] demonstrated that the serum levels of testosterone were significantly lower in Per1/Per2‐knockout mice than in wild‐type mice. The underlying mechanism through which the circadian rhythm regulates testosterone secretion may involve the activation of the PKA–steroid acute regulatory protein signaling pathway [71]. Moreover, research has shown that sleep deprivation can reduce testosterone levels and that the recovery of testosterone levels is impaired in old rats compared with young rats [72]. Although these findings indicate the neurohumoural pathway through which circadian rhythm impacts tissue metabolism and deterioration, additional research is needed to establish more direct evidence.

### 3.3 Immune and Oxidative Stress Responses

Inflammatory signals and immune function are intricately involved in normal physiology and can be disrupted with aging [73]. It has been revealed that immune response are involved in regulating tissue repair [74, 75]. Most immune cells, such as monocytes, macrophages, natural killer cells, master cells, neutrophils, and lymphocytes, express clock genes within a period of approximately 24 h, which has noticeable impacts on cellular activities, including a daily rhythm of migration, proliferation, phagocytosis, and secretion [76, 77, 78, 79]. For example, in an inflammatory environment, the production of inflammatory factors, including IL‐1β, IL‐6, IL‐18, and Ccl2, by bone marrow‐derived macrophages is attenuated by Bmal1 and REV‐ERBα expression, indicating the anti‐inflammatory function of these two proteins [80, 81, 82]. Disruption of the circadian rhythm is accompanied by elevated levels of inflammatory cytokines, including IL‐6, TNF‐α, and IFN‐γ [83, 84]. Restoring the circadian rhythm with melatonin effectively reduces the inflammatory response [85]. Aging is related to the loss of circadian rhythmic innate immune responses [86]. Kruppel‐like factor 4 (Klf4) is considered an important transcription factor that regulates macrophage polarization and function [87]. Blacher et al. [88] reported that the diurnal expression of Klf4 was lower in aged macrophages than in young macrophages and that the loss of Klf4 expression was accompanied by circadian rhythm disruption in the phagocytic activity of macrophages, indicating that the circadian rhythm was associated with aging‐related changes in macrophage phagocytosis. Moreover, the production of the antiosteoclastogenic factor IL‐10, which is secreted by regulatory T cells, is diminished when the circadian clock is disrupted [89]. Thus, regarding the effects of the immune response on tissue deterioration and the roles of circadian clock genes in immune cells, inflammatory modulation may be a potential mechanism by which the circadian rhythm regulates tissue deterioration. However, there is still a lack of relevant research that focuses on the effects and regulatory mechanism of clock genes and their downstream target genes on the tissue immune microenvironment, which needs to be carried out in the future.

The circadian rhythm has also been shown to regulate redox homeostasis. A study involving 66 participants revealed increased activity of superoxide dismutase (SOD) and glutathione peroxidase (GPX), along with an increase in the glutathione concentration noted at night, indicating the influence of the circadian rhythm on oxidative stress [90]. Compared with normal individuals, ischemic stroke patients presented lower levels of SOD and Bmal1 [91]. The clock gene Per1 coordinates with the enzyme GPX1, influencing mitochondrial dynamics in alignment with circadian rhythms and oxidative stress [92]. Under pathological conditions, such as those arising from shift work, disruptions in circadian rhythms can lead to a decline in antioxidant capacity, resulting in the accumulation of reactive oxygen species (ROS) that damage DNA and proteins, thereby exacerbating conditions such as cardiovascular and chronic pulmonary diseases [93, 94]. These findings underscore the importance of maintaining oxidative balance through the regulation of circadian rhythms as critical targets for the prevention and treatment of diseases related to oxidative stress.

### 3.4 Tissue Repair

Mesenchymal stromal cells (MSCs) are adult stem cells that can migrate into damaged areas to maintain tissue homeostasis through proliferation, osteogenic pluripotency, and immunomodulation [95]. MSCs also express clock genes in a circadian pattern [96, 97]. Rhythmic expression of the clock genes Bmal1, Per2, and Nr1d1 in MSCs has also been observed in vitro [98]. The biological clock regulates hormone signals and transcription factors in MSC fate decisions. It also engages with noncoding RNAs and epigenetic modifiers and plays a role in the remodeling of chromatin [99]. MSC proliferation and pluripotency are reportedly related to Bmal1 expression [100]. Inhibition of RORα, an upstream regulator of Bmal1, was able to reduce MSC pluripotency [101]. Additionally, it has been reported that MSCs isolated from Bmal1‐deficient mice have a reduced capacity for differentiation [102]. However, Zhou et al. [103] reported the opposite findings, suggesting that Bmal1 negatively influences the pluripotency of MSCs. Their study revealed that silencing Bmal1 expression increased the differentiation capability of MSCs [103]. These discrepant conclusions may be due to the use of different cell isolation methods, cell culture conditions or cell model systems. In addition, a limitation of these studies is that most of the experiments were performed in mouse MSCs rather than human MSCs. More research on the expression and role of Bmal1 in human MSCs is warranted. Furthermore, all these studies were performed in vitro; thus, they cannot recapitulate the complicated microenvironment of MSCs in vivo. Thus, more in vivo experiments must be performed in the future.

The vasculature delivers various molecular signals to cells during tissue development, remodeling, and regeneration [104]. Many studies have shown that the circadian rhythm plays important roles in maintaining vascular homeostasis [105, 106]. The mechanisms of angiogenesis under the control of the circadian clock have been determined in part. Xu et al. [107] reported that decreased expression of Bmal1 could inhibit endothelial cell activities, including proliferation, migration, and vascular network formation, by downregulating VEGF expression. In vivo experiments further confirmed that Bmal1 deletion resulted in impaired angiogenesis in mice [107]. The clock gene Cry2 is involved in lipid metabolism and vascular aging, primarily by influencing phospholipid metabolism and Serac1 during endothelial senescence [108]. Similarly, Wang et al. [109] investigated the effect of the clock gene Per2 on endothelial function and reported that endothelial cells from Per2 mutant mice exhibited activated Akt signaling, increased senescence, and impaired proliferation and tube formation abilities. When subjected to hind‐limb ischemia, Per2 mutant mice presented decreased blood flow and developed autoamputation in the distal limb, indicating that Per2 deficiency could significantly impair endothelial cell function and accelerate vascular senescence, thus leading to endothelial dysfunction [109]. Furthermore, Mastrullo et al. [110] demonstrated that pericytes, a perivascular cell population involved in the regulation of endothelial cell function and vascular maturation, also express clock genes in a circadian pattern. They reported that direct coculture of pericytes and endothelial cells could induce a circadian rhythm in endothelial cells [110]. It has been demonstrated that diurnal variation in circulating endothelial nitric oxide synthase (NOS3) levels, which regulate both endothelial functions, is established in healthy women, indicating that NOS3 may be another mediator of circadian rhythm‐regulated vessel formation [111, 112, 113]. More effort is still needed to directly verify the protective effects of circadian clock‐regulated endothelial function on tissue repair in the future.

### 3.5 Autophagic Flux

Autophagy (macroautophagy, microautophagy, and chaperone‐mediated autophagy) is an evolutionarily conserved catabolic process that is activated in response to cellular stress in which cells digest themselves to promote cell survival [114]. Autophagy targets damaged organelles, misfolded proteins, and intracellular microbes and plays a pivotal role in maintaining homeostasis, inhibiting apoptosis and preventing the senescence of multiple organs [115]. Ma et al. [116] demonstrated that autophagy activity is significantly attenuated in aged MSCs compared with young MSCs. The inhibition of autophagy by 3‐methyladenine could increase ROS and p53 levels and accelerate MSC senescence. Furthermore, they reported that the activation of autophagy could restore the impaired biological functions of aged MSCs [116]. These findings highlight the critical role of autophagy in tissue deterioration, and the activation of autophagy can partially reverse this deterioration. Recent research has also shown that autophagy activity is dependent on the circadian rhythm. The expression of various autophagy‐related genes, such as UNC51‐like kinase 1 (Ulk1), microtubule‐associated protein 1 light chain 3 B (LC3B), GABA (A) receptor‐associated protein‐like 1 (Gabarapl1), and BCL2/adenovirus E1B‐interacting protein 3 (Bnip3), is regulated by the circadian rhythm [117]. Disruption of the circadian clock results in loss of rhythmic chaperone‐mediated autophagy activity and significant changes in the chaperone‐mediated autophagy‐dependent cellular proteome [118]. These findings suggest that the regulation of autophagy by the circadian clock may have positive effects on reversing tissue deterioration.

Mitophagy, a selective autophagic process that recognizes and degrades damaged mitochondria, plays a key role in maintaining mitochondrial homeostasis and functionality [119]. Mitochondrial dysfunction is involved in tissue deterioration [120]. Recently, evidence of an interaction between circadian rhythm and mitophagy has emerged [121]. Li et al. [122] confirmed that Bmal1 knockout directly reduces mitophagy and causes mitochondrial dysfunction. The effects of Bmal1 on mitochondrial activity may occur through regulation of the expression of Bnip3 [122]. Whether mitophagy is involved in the process by which the circadian clock regulates tissue deterioration remains to be further investigated.

In summary, the underlying mechanisms through which circadian rhythm disruption impacts tissue deterioration have been partially elucidated. However, further research is necessary to fully elucidate the pathological changes in tissues associated with circadian rhythm disruption. The relationship between circadian rhythm disruption and CDDs will be discussed in the next section.

## 4 Circadian Disruption in Disease Pathogenesis: Mechanisms and Targets

**FIGURE 3:**
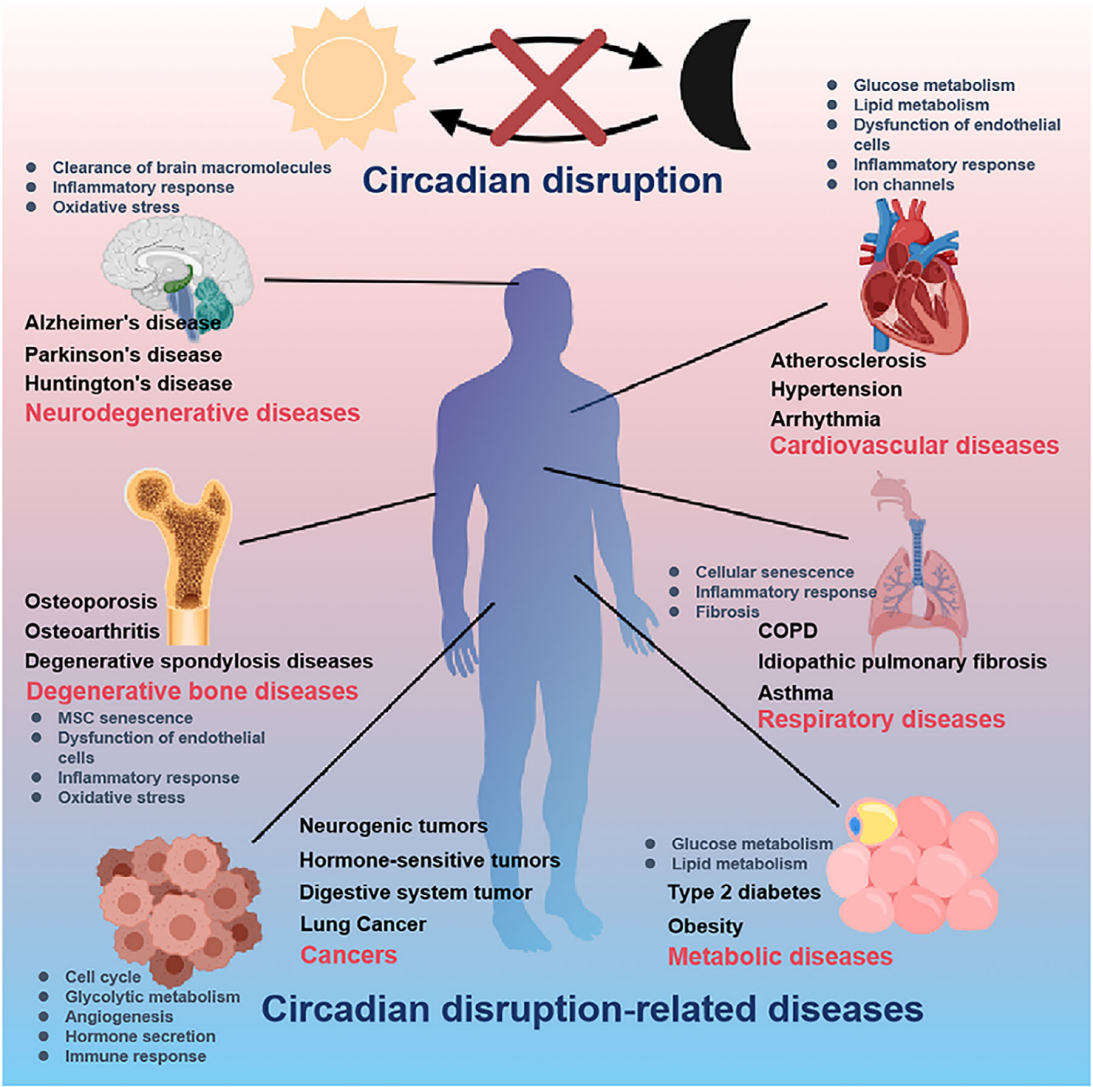
Impact of circadian rhythm disruption on aging‐related diseases. Circadian rhythm disruption can result in the development of aging‐related diseases, such as bone degenerative diseases, neurodegenerative diseases, cardiovascular diseases, metabolic diseases, respiratory diseases, and cancers.

The circadian rhythm is an important regulatory factor in bone health and diseases. Disruption of the circadian rhythm can lead to CDDs such as degenerative diseases, cardiometabolic disorders, and cancers (Figure 3). A deeper understanding of the role of the circadian rhythm in bone deterioration will be conducive to the development of therapeutics for CDDs.

### 4.1 Degenerative Diseases

#### 4.1.1 Bone Degenerative Diseases

**FIGURE 4:**
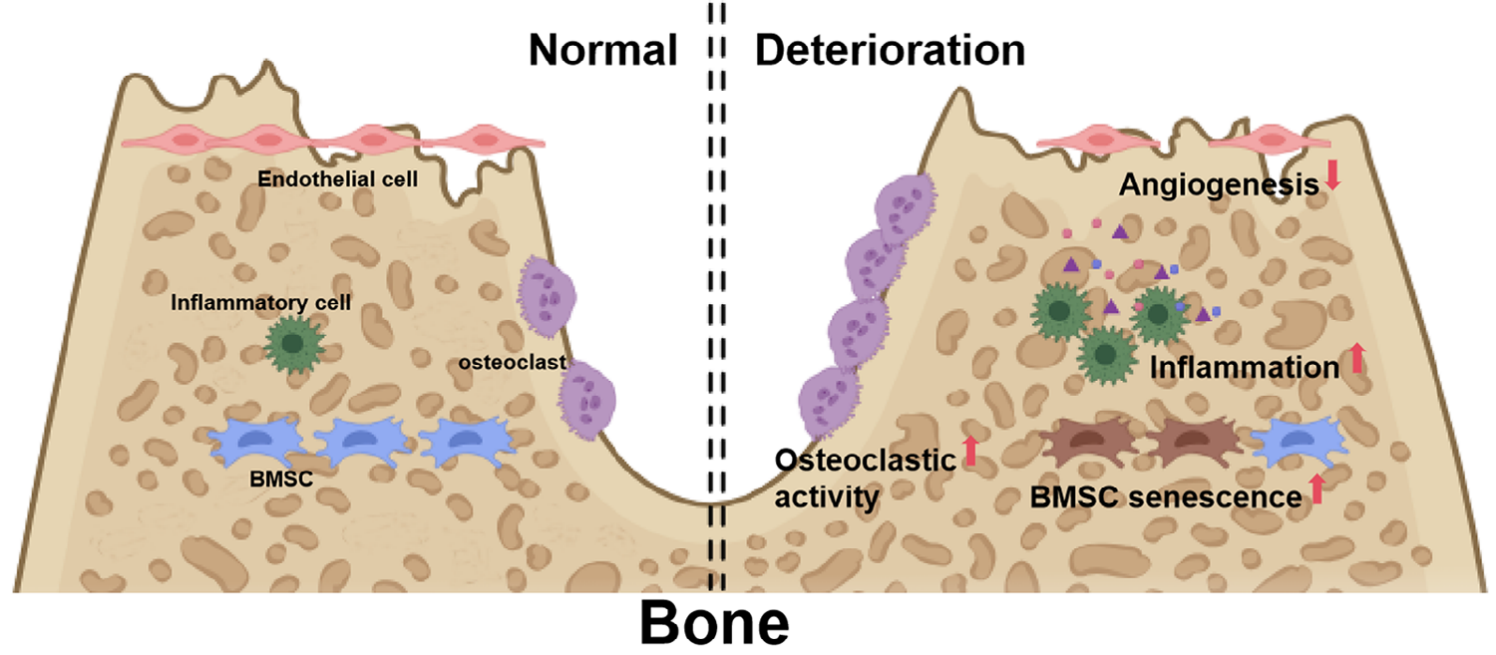
Regulation of bone deterioration. The process of bone deterioration is regulated by multiple cell types, including osteoclasts, endothelial cells, BMSCs, and inflammatory cells.

Bone deterioration is controlled by various cell types (Figure 4), such as osteoclasts, osteoblasts, osteocytes, bone marrow‐derived MSCs (BMSCs), and inflammatory cells [123, 124]. In recent years, numerous studies have shown that the circadian rhythm influences bone remodeling and the progression of bone degenerative diseases (Table 1). The underlying mechanism and treatment of bone degenerative diseases have become a research hotspot in recent years [125, 126]. With the increasing problem of an aging global population, the incidence of osteoporosis has gradually increased over the past few decades, placing a significant burden on human society [127, 128]. In addition to the widely studied effects of estrogen, nutritional status, and genetic or physical factors on osteoporosis [129], both later sleep onset and decreased sleep quality have been identified as risk factors for osteopenia and osteoporosis [130, 131, 132, 133]. To investigate the association between night shift work and bone mineral density (BMD), Kim et al. [134] conducted a cross‐sectional study involving 3005 individuals (aged 18–50 years) and reported that the mean BMD of workers with nondaytime shifts was significantly lower than that of daytime workers, indicating that the risk for osteopenia was increased in workers with circadian rhythm disruption. Similarly, Xu et al. [135] evaluated the effects of chronic sleep deprivation on bone metabolism and bone mass in rats. They demonstrated that BMD, bone volume, trabecular bone thickness, trabecular bone number, and the levels of bone turnover biomarkers were significantly lower in rats with chronic sleep deprivation than in those with normal sleep [135]. These results are consistent with the findings of Schilperoort and colleagues, who reported that circadian disruption can result in altered trabecular bone structure and reduced levels of bone resorption markers (serum tartrate‐resistant acidic phosphatases) and bone formation markers (serum PINP) in mice [136]. However, Swanson et al. [137] used a model of sleep restriction combined with circadian rhythm disruption that mimicked shift work and argued that circadian disruption in humans can lead to a decrease in the level of a bone formation marker (serum PINP) without a change in the level of a bone resorption marker (serum NTX). The different results might be due to different intervention intensities and times, sample sizes and species [137]. Nevertheless, circadian disruption is detrimental to bone metabolism and bone mass and may lead to osteoporosis; therefore, circadian rhythm components are potential therapeutic targets in osteoporosis.

**TABLE 1:** Relationships between circadian disturbances and bone degenerative diseases.

Bone degenerative diseases	Circadian disturbances	Species	Subjects	Test periods	Results	Ref
osteopenia	decreased sleep quality and later sleep timing	human	915 participants (aged 45‐65 years old, 56% women)	NA	declined sleep quality and later sleep timing were associated with osteopenia	[130]
osteoporosis	short and long sleep patterns	human	2637 individuals at different age	NA	both short and long sleep patterns were more prevalent among individuals with osteoporosis	[131]
osteopenia	shift working	human	3005 workers (aged 18‐50 years old)	NA	other‐than‐daytime workers had significant lower mean BMD of the total femur and lumbar spine, decreased serum 25‐hydroxyvitamin D levels, and higher prevalence of osteopenia compared to daytime workers	[134]
decreased bone turnover	chronic sleep deprivation	rat	24 rats were randomly divided into chronic sleep deprivation and control groups	3 months	rats with chronic sleep deprivation had significant decreased BMD, bone volume, trabecular bone thickness, trabecular bone number and BTMs such as serum NTX and PINP compared to rats with normal sleep	[135]
altered bone turnover and structure	shift in light‐dark cycle	mouse	13 to 17‐week‐old female APOE*3‐Leiden.CETP mice	15 weeks	shift in light‐dark cycle affected both trabecular bone structure and bone resorption marker (serum tartrate‐resistant acidic phosphatase) and bone formation marker (serum PINP)	[136]
altered bone turnover	sleep restriction	human	6 young men (aged 20‐27 years old) and 4 elderly men (aged 55‐65 years old)	3 weeks	bone formation marker (serum PINP) was decreased but bone resorption marker (serum NTX) was unchanged after treatment with sleep restriction.	[137]
OA	dark‐light cycle disruption	rat	60 adolescent (6‐8 weeks) male SD rats	6 weeks	decreased BMD, BV/TV, and trabecular number, and increased ratio of the Rankl/OPG and activation of the mononuclear/phagocyte system were seen in hindlimbs of OA rats with dark‐light cycle disruption	[142]
OA	sleep rhythm disturbance	rat	180 8‐week‐old Wistar rats	4‐8 weeks	sleep rhythm disturbance downregulated the Bmal1 expression and upregulated IL‐6 level, leading to temporomandibular joint OA development.	[143]
IVD degeneration	*Bmal1* deficiency	mouse	6‐month‐old mice	6 months	Loss of Bmal1 leaded to age‐dependent IVD degeneration, as shown by signs of disorganisation, fibrosis, calcification and narrowing of spaces.	[151]
IVD degeneration	*Bmal1* deficiency	rat	IVD specimens from 8‐week‐old SD rats	14 days	targeted deletion of Bmal1 resulted in increased inflammatory response, oxidative stress, and apoptosis of nucleus pulposus cells by reducing Nrf2 expression	[152]

Abbreviations: Bmal1, brain and muscle ARNT‐like protein‐1; BMD, bone mineral density; BTMs, bone turnover markers; BV/TV, percent bone volume with respect to total bone volume; IVD, intervertebral disc; NA, not applicable; Nrf2, nuclear factor erythroid kmk2‐related factor 2; NTX, N‐terminal cross‐linked telopeptide of type I collagen; OA, osteoarthritis; OPG, osteoprotegerin; PINP, N‐terminal propeptide of type I procollagen; Rankl, receptor activator of nuclear factor κB ligand.

Recently, circadian rhythm disruption was found to be related to the progression of osteoarthritis (OA) [138]. The circadian rhythm in cartilage is considered an important regulatory mechanism for tissue homeostasis and OA development [139]. The circadian rhythm in murine cartilage decreases with age during OA development [140]. Song et al. [141] reported that Bmal1 knockdown in chondrocytes resulted in significantly decreased expression of aggrecan and COL2A1 and increased expression of proinflammatory factors, such as MMP‐3 and MMP‐13, via activation of the Wnt/β‐catenin signaling pathway. Furthermore, they reported that light/dark cycle shift‐induced circadian rhythm disturbance significantly enhanced cartilage matrix degradation and synovial inflammation, suggesting that circadian rhythm disturbance is a risk factor for OA progression [141]. Moreover, circadian rhythm disorders induced by dark‒light cycle disruption significantly disrupted bone metabolism and destroyed the structure of subchondral bone in the hindlimbs of OA rats, as evidenced by decreased BMD, percent bone volume with respect to total bone volume, and trabecular number, an increased ratio of the nuclear factor κB ligand (Rankl)/osteoprotegerin and activation of the mononuclear/phagocyte system in the bone marrow [142]. Chen et al. [143] confirmed that sleep rhythm disturbance could induce downregulated expression of Bmal1 and the development of temporomandibular joint OA. Depletion of Bmal1 resulted in increased expression of IL‐6 and activation of the MAPK/ERK pathway. These results provide insight into the molecular mechanism through which the circadian rhythm affects OA development [143]. Cry2 is also reported to be dysregulated in aging‐related OA. Compared with wild‐type mice, Cry2 knockout mice with experimental OA presented significantly increased severity in the cartilage, subchondral bone, and synovium [144]. In addition, circadian rhythm disruption can lead to the upregulation of Per1–GSK3β/β‐catenin signaling in mandibular condylar chondrocytes, resulting in increased expression of cartilage matrix‐degrading enzymes and the development of temporomandibular joint OA [145]. Interestingly, Sumsuzzman et al. [146] demonstrated that the regulation of circadian rhythms with melatonin treatment could maintain the anabolic‒catabolic balance during OA development, providing a regulatory mechanism linking melatonin to the circadian rhythm and treatment for OA. These results suggest that circadian rhythm disruption affects chondrocyte behavior and subchondral bone, thus leading to the development of OA.

As a leading cause of adult spinal cord dysfunction, degenerative spondylosis diseases (DSDs) are related to various age‐related pathologies, including calcification and hypertrophy of the intervertebral disc (IVD), ligaments, and osseous tissues. In recent decades, increasing research has led to drastic advances in understanding the pathophysiology, diagnosis, and treatment of DSDs [147]. Studies have shown that circadian rhythm disruption can contribute to the development of DSDs [148, 149]. Recently, increasing evidence has confirmed the existence of a circadian rhythm within the IVD, and disruption of the circadian clock could lead to accelerated IVD aging and degeneration [150, 151]. The underlying mechanism by which circadian rhythm impacts the development of IVDs has also been partly revealed. Peng et al. [152] reported that the circadian rhythm was abolished in degenerated human and rat nucleus pulposus tissues. They also demonstrated that Bmal1 deficiency resulted in increased inflammation, oxidative stress, and apoptosis in nucleus pulposus cells by reducing the expression of nuclear factor erythroid kmk2‐related factor 2 [152]. In addition, Jiang et al. [117] reported that limited autophagy protected against IVDs, but excessive autophagy could be induced by disrupted circadian rhythm and thereby accelerate IVD degeneration. However, further investigations are still needed to fully elucidate the molecular mechanism through which the circadian rhythm regulates autophagy during IVD degeneration [117]. Accordingly, restoring the dampened circadian clock by inhibiting the RhoA/ROCK pathway with Y‐27632 or melatonin could protect against compression‐induced IVD degeneration [153]. Since an interaction between circadian rhythm and IVD degeneration has already been found, the regulation of the circadian clock could partially reverse tissue aging and potentially serve as an effective clinical approach to mitigating IVD degeneration. Furthermore, the relationships between circadian rhythm and other DSDs, such as spinal stenosis and lumbar spondylolisthesis, remain unstudied and warrant future investigation.

#### 4.1.2 Neurodegenerative Diseases

Alzheimer's disease (AD), identified by the buildup of amyloid plaques along with neurofibrillary tangles within the brain, has emerged as the leading cause of dementia and significantly impacts global health. In addition to genetic influences, the onset of AD is shaped by numerous risk factors, such as aging, systemic inflammation, environmental influences, and lifestyle choices [154]. Circadian disruption has been observed in AD patients [155]. Sleep disorders are regarded as both potential risk factors for and consequences of AD, indicating a reciprocal relationship between circadian disturbances and AD [156]. Circadian disruption has been reported to hinder the clearance of brain macromolecules (such as β‐amyloid and tau proteins associated with microtubules) through the glymphatic–vascular–lymphatic system, increase oxidative stress within the brain, and reduce the levels of circulating melatonin [157].

Parkinson's disease (PD) is another prevalent neurodegenerative disorder that is associated with disturbances in circadian rhythms [158]. Significant links have been established between circadian disruption and PD, which can lead to adverse effects on homeostasis and potentially worsen the progression of the disease [159]. Compared with non‐PD older adults, PD patients are more likely to face sleep‐related disturbances [160, 161]. Downregulated expression of *Bmal1* was observed in SNCA^A53T^ mice, a model for PD [162], suggesting that clock genes may be altered in PD. Bioinformatic analysis further revealed that circadian rhythm‐related genes, including *AK3*, *RTN3*, and *LEPR*, can be used as biomarkers to distinguish PD samples from control samples [163].

Circadian disruption also coexists with Huntington's disease (HD). Compared with normal individuals, HD patients exhibit a delayed sleep phase and worse sleep quality [164, 165]. In a Drosophila model of HD, changes in the expression of clock genes, including *Period* and *Timeless* genes, were observed. When melatonin or curcumin was administered via food, the regular expression patterns of *Period* and *Timeless*, as well as locomotion ability and eclosion behavior, were restored [166]. Reports show that poor sleep can occur even in the early stages of the disease, including in asymptomatic carriers of the HD mutation [167]. These findings underscore the importance of prompt diagnosis and proactive management of sleep disturbances in individuals affected by HD.

Overall, these findings contribute to a new understanding for future clinical assessments of neurodegenerative disorders and establish a basis for additional investigations into how circadian rhythm‐related processes may influence the progression of these diseases.

### 4.2 Cardiometabolic Disorders

#### 4.2.1 Cardiovascular Diseases

Cardiovascular disease has become the most common cause of death worldwide. It has been demonstrated that circadian rhythm is a risk factor for cardiovascular diseases [168]. The biological clock modulates many cardiovascular variables, such as heart rate, heart rate variability, electrocardiogram, blood pressure, and endothelial function. Numerous clinical and experiential investigations have emphasized that disturbances in circadian rhythms may ultimately result in dysfunctional cardiovascular performance [10, 169]. Increasing evidence shows that the circadian rhythm plays a crucial role in various aspects of atherosclerosis, such as glucose metabolism, lipid metabolism, dysfunction of endothelial cells, the phenotype of vascular smooth muscle cells, and immune inflammatory responses [170]. Schilperoort et al. [171] reported that APOE*3‐Leiden.CETP mice exposed to alternating light‒dark cycle‐induced circadian disruption presented a notable increase in atherosclerosis, with a nearly twofold increase in the size and severity of lesions [171]. Xie et al. [172] reported that the absence of *Bmal1* led to activated NF‐κB signaling, increased oxidative stress, and an increased inflammatory response in human aortic endothelial cells, thus accelerating cardiovascular diseases associated with atherosclerosis [172]. The results of bioinformatic analysis revealed that two circadian regulatory genes, CCR1 and C3AR1, are related to immune infiltration and the progression of atherosclerosis [173]. However, the precise mechanisms by which they contribute to the atherosclerotic process are still not fully understood.

Circadian disruption is associated with a heightened risk of hypertension. Elevated blood pressure and low inflammatory status are linked to inadequate sleep and shift work [174]. Recent studies have suggested that the peripheral clocks present in smooth muscle, perivascular adipose tissue, liver, adrenal glands, and kidneys play a role in regulating blood pressure [175].

The relationship between circadian disruption and arrhythmia has also been investigated. The existing knowledge regarding the impact of circadian disruption on cardiac electrophysiology has been analyzed, emphasizing the control of repolarization and ion channels [176]. Jeyaraj et al. [177] demonstrated that an imbalance in Klf15 levels, which serves as a clock‐dependent oscillator, leads to the disruption of rhythmic QT variation, irregular repolarization, and increased vulnerability to ventricular arrhythmias. In another study, Schroder et al. [178] established inducible cardiomyocyte‐specific deletion of *Bmal1* in mice and reported that the loss of Bmal1 in cardiomyocytes resulted in a disrupted circadian pattern of *the* expression of Kcnh2, a K(+) channel gene, leading to prolonged ventricular repolarization. Nonetheless, these findings offer important insights for identifying potential therapeutic targets for treating circadian rhythm disruption‐related cardiovascular diseases.

#### 4.2.2 Respiratory Diseases

Chronic obstructive pulmonary disease (COPD) is a global health problem that is characterized primarily by persistent airflow limitation and progressive dyspnea. A prospective cohort study revealed a circadian rhythm in the manifestation of COPD symptoms, with a greater proportion of patients experiencing respiratory symptoms in the morning and daytime than in the night [179]. Genetic research suggests that circadian rhythms, including cellular senescence and inflammatory responses, are involved in the pathology of COPD [180]. One clinical study demonstrated significantly reduced expression of clock proteins—including Bmal1, Per2, Cry1, and REV‐ERBα—in the lung tissue of COPD patients compared with controls [181]. Additionally, an animal study revealed altered clock gene expression in mice exposed to tobacco smoke [182]. These findings suggest that circadian rhythm disruption may contribute to the progression of COPD.

Idiopathic pulmonary fibrosis is a disease histologically characterized by the formation of fibroblast foci in the lung parenchyma. Recent research has revealed a significant link between the progression of fibrosis and the expression of clock genes. Studies have shown that REVERBα in fibroblasts suppresses the development of pulmonary fibrosis and that deletion of its DNA‐binding domain leads to exacerbated fibrosis [183]. Conversely, Bmal1 knockdown inhibits the differentiation of normal human lung fibroblasts into myofibroblasts [184]. These findings suggest that clock genes such as REVERB and Bmal1 are involved in fibrotic transformation.

Asthma, a common respiratory disease, has been shown to be influenced by circadian rhythm variations in terms of severity, with studies indicating that this effect is independent of daily behaviors and environmental changes [185]. Serum cortisol, widely used as an anti‐inflammatory marker in inflammatory diseases, was found to be lower in asthma patients than in controls in one clinical study, with this difference being more pronounced at midnight [186]. Furthermore, several clinical studies suggest that administering corticosteroid medications in the evening is more effective at alleviating asthma symptoms at night than at other times [187, 188, 189]. These findings indicate that the circadian rhythm of cortisol is involved in the onset of asthma symptoms. Genetic‐level studies have revealed altered expression of circadian clock genes in asthma patients [190].

In summary, disruption of pulmonary circadian rhythms can lead to alterations in lung function characteristic of chronic lung diseases, including increased airway inflammation and hyperresponsiveness, oxidative stress, and cytokine responses. Future research focusing on the genetic aspects of circadian rhythms will contribute to a deeper understanding of disease progression and provide new therapeutic perspectives.

#### 4.2.3 Metabolic Diseases

Type 2 diabetes mellitus is the most prevalent metabolic disorder in humans and is characterized by insulin resistance and dysfunction of pancreatic β‐cells. Numerous circadian misalignments, including changes in light‒dark cycles, sleep‒wake patterns, rest‒activity schedules, fasting‒feeding intervals, shift work, evening chronotypes, and social jetlag, as well as mutations in clock genes, may play a role in the onset of diabetes and the management of glycemic control in individuals with this condition [191]. Considering the simultaneous high rates of type 2 diabetes and disturbances in circadian rhythms, gaining insight into the mechanisms that drive the effects of circadian disruption on glucose metabolism could help improve type 2 diabetes control. The hypothalamic‒pituitary‒adrenal axis, which plays a vital role in regulating glucose metabolism and affecting the progression of T2D, is regulated by the circadian clock [192]. In a multicenter study involving 68 subjects, researchers reported a positive correlation between circadian disruption and endogenous hypercortisolism [36]. Circadian disruption may also affect glucose tolerance by decreasing β‐cell function and insulin sensitivity [193]. Individuals with impaired β‐cell function tend to experience longer durations of night shifts [194]. Inadequate sleep and disruption of circadian rhythms led to a reduction in the resting metabolic rate of individuals but also elevated plasma glucose levels following a meal, a consequence stemming from insufficient secretion of insulin by the pancreas [195].

Preliminary evidence has indicated that circadian disruption may be a risk factor for abnormal lipid metabolism and the development of obesity [196]. A randomized crossover study revealed that chronic shift workers exhibit increased levels of acylated ghrelin due to circadian misalignment, even when dietary intake is meticulously regulated [197]. Aggarwal et al. [198] reported that the deletion of Per3 led to increased adipogenesis in vivo through a pathway involving clock output, in which both Per3 and Bmal1 directly influence the expression of Klf15. These results indicate that Per3 plays a significant role in the APC clock and modulates adipogenesis in vivo [198]. Shostak et al. [54] demonstrated that mice with circadian clock mutations exhibit low and arrhythmic levels of FFAs and glycerol in their bloodstream, alongside reduced lipolysis rates and heightened sensitivity to fasting. In contrast, disruptions in circadian rhythms contribute to the buildup of TGs in white adipose tissue, resulting in increased fat accumulation and hypertrophy of adipocytes [54]. Additionally, implementing time‐restricted feeding helped protect mice from significant weight gain and metabolic disorders [199], suggesting that circadian rhythms could serve as promising targets for obesity management.

### 4.3 Cancers

#### 4.3.1 Neurogenic Tumors

The literature suggests that circadian rhythm disruption contributes to the transformation of normal tissue to tumor tissue and that tumor tissue can exploit abnormal biological rhythms to meet its metabolic demands [200]. The impact of the circadian rhythm on tumors is systemic. Independent of specific cancer types, the core clock gene Bmal1 governs the cell proliferation rhythm essential for tumor angiogenesis by regulating the cell cycle genes CCNA1/CDK1. Disruption of this rhythm can significantly inhibit tumor growth [201]. Rhythm disruption promotes transformation by driving the loss of APC heterozygosity, which excessively activates Wnt signaling and MYC gene expression, consequently enhancing glycolytic metabolism, which is essential for sustaining tumor growth [202]. Glioblastoma multiforme cells actively exploit the circadian clock to drive their malignant progression. In glioblastoma stem cells, the clock protein Clock enhances POSTN secretion via the OLFML3–HIF1α axis. In turn, POSTN activates the TBK1 pathway in vascular endothelial cells, significantly promoting tumor angiogenesis. This Clock–POSTN–TBK1 signaling axis, which is directly associated with poor patient prognosis, represents a potential therapeutic target [203]. Other types of neurogenic tumors are also closely linked with circadian rhythms. For example, in pituitary tumors, systemic circadian disruption (e.g., shift work) can accelerate tumor growth. The underlying molecular mechanism involves the dysregulation of core clock genes, particularly the significant upregulation of the Per2 protein, which interacts with HIF‐1α and leads to the upregulation of key downstream cell cycle genes (e.g., Ccnb2, Cdc20, Espl1), ultimately promoting cell cycle progression and inhibiting apoptosis [204].

#### 4.3.2 Hormone‐Sensitive Tumors

The circadian rhythm plays a complex and critical role in the development and progression of hormone‐sensitive tumors. Abnormal light exposure can disrupt the rhythmic expression of core clock genes, such as Bmal1 and Aanat, which in turn suppresses the secretion of hormones such as melatonin and progesterone. This hormonal imbalance ultimately activates the PKC‐α/Akt signaling pathway, collectively driving angiogenesis and endometrioid adenocarcinoma formation [205]. In breast cancer, the transcription factor FOXK1 serves as a crucial hub connecting metabolic disorders to the core clock machinery in cancer cells. Insulin resistance enhances FOXK1 function at both the transcriptional and posttranslational levels by upregulating O‐linked N‐acetylglucosamine transferase. Nuclear FOXK1 then recruits corepressor complexes to specifically bind and silence core clock genes, including Clock, Per2, and Cry2, leading to uncontrolled cell proliferation and driving the malignant progression of breast cancer [206]. In addition, the PRMT6/PARP1/CRL4B complex specifically silences the core clock gene Per3. This disruption directly leads to cellular circadian disturbances, thereby promoting the proliferation and invasion of breast cancer [207]. The binding of the clock protein RORA to HDAC3 can suppress the expression of the immune checkpoint molecule PD‐L1, thereby enhancing antitumor immunity [208]. In tumors such as melanoma, the RNA helicase DDX3X competitively binds to RORA, preventing its inhibitory effect on PD‐L1 and leading to PD‐L1 upregulation and immune evasion [208]. This molecular competition mechanism not only affects tumor prognosis but also enables the development of a scoring system based on the expression levels of these molecules to predict the response to immunotherapy.

#### 4.3.3 Other Tumors

The core clock genes Clock and Bmal1 directly regulate the expression of the interleukin‐20 receptor subunit beta (IL‐20Rβ), thereby enhancing the responsiveness of tumor cells to IL‐20 and activating the critical JAK/STAT prosurvival pathway. This mechanism is essential for preserving the viability of leukemia‐initiating cells [209]. Furthermore, the Per gene is a prognostic marker in lung cancer, with its high expression predicting better survival outcomes. Metabolic interventions such as glucose restriction can upregulate Per expression by activating the AMPK‒SIRT1 pathway and restoring the gene rhythms disrupted by tumors, revealing a bidirectional role in which restoring the circadian rhythm can suppress cancer [210]. Research has also confirmed that Per1, as a target of miR‐34a, is involved in suppressing the growth and invasion of cholangiocarcinoma. Reduced Per1 expression tends to lead to malignant phenotypes in cholangiocarcinoma [211]. Additionally, circadian rhythms can be used to predict disease occurrence and patient prognosis. A cohort study suggested that nighttime light exposure, a recognized disruptor of circadian rhythms, may be a risk factor for pancreatic ductal adenocarcinoma [212]. Simultaneously, circadian rhythms can be quantified as clinical data and are associated with survival rates in patients with metastatic colorectal cancer [213]. Colorectal cancer patients with robust diurnal rest‒activity rhythms exhibit better health‐related quality of life during survival [214].

In summary, circadian rhythms are intricately linked to tumor development, with disruptions in these rhythms correlating with an increased incidence and malignancy of various tumors. A more profound understanding of the regulation of circadian rhythms across different tumor types could pave the way for innovative therapeutic strategies, offering a novel perspective on cancer treatment.

## 5 Emerging Therapeutic Strategies Based on Circadian Rhythm Regulation

**FIGURE 5:**
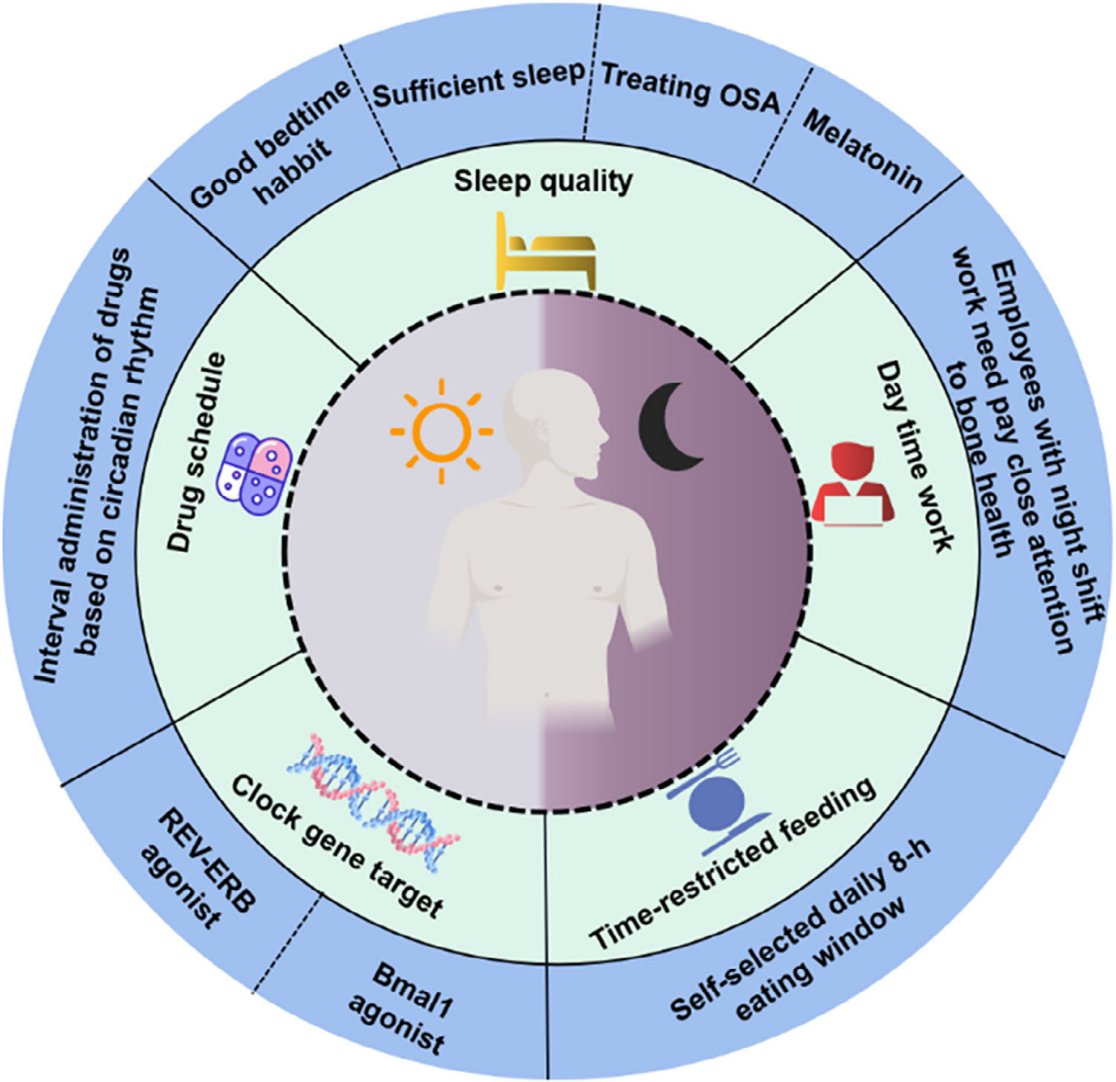
Convergence of methods that are proposed to improve human health. Several behavioral or pharmacological interventions can be used to improve human health, including sufficient sleep, daytime work, time‐restricted feeding, clock gene targeting, and drug schedules.

The relationship between circadian rhythm and tissue homeostasis offers new insights into improving therapies for CDDs (Figure 5). Biological rhythm therapy, as an emerging intervention strategy for various diseases, enhances health outcomes by precisely regulating the human circadian rhythm system. This approach provides innovative solutions for the treatment of diverse medical conditions. In the following sections, we introduce three types of therapies that are based on circadian rhythm regulation: pharmacological modulators, chrono‐nanomedicine, and nonpharmacological interventions. Additionally, we discuss current translational challenges and future directions in this field.

### 5.1 Pharmacological Modulators

Melatonin is effective in improving sleep and has been used to treat several sleep disorders [215, 216]. It has been demonstrated that melatonin is involved in regulating the tissue microenvironment [217, 218, 219, 220]. The underlying mechanisms through which melatonin regulates circadian rhythm and tissue deterioration have been reported in a recent study from our group. We found that melatonin receptor 1 could activate AMPKβ1 phosphorylation, which subsequently triggered Bmal1 expression and modulated endochondral bone formation by increasing cell proliferation and matrix synthesis [221].

Since disruption of the circadian rhythm can affect tissue degeneration and result in CDDs, some drugs targeting clock genes may be beneficial for human health and thus deserve broader investigation and development. Several drugs and small molecules have been developed to treat metabolic diseases in animal models through the modulation of circadian rhythm. For example, Solt et al. [222] synthesized a REV‐ERB ligand that can alter core clock gene expression in the hypothalamus and metabolic gene expression in peripheral tissues, including the liver, adipose tissue, and skeletal muscle, in vivo. After administration to diet‐induced obese mice, the REV‐ERB agonist resulted in increased energy expenditure, decreased obesity, and improved dyslipidemia and hyperglycemia [222]. Song et al. [223] demonstrated that the intraperitoneal administration of SR9009, another REV‐ERB agonist, prevents osteoclast differentiation and bone resorption, resulting in inhibited ovariectomy‐induced bone loss in mice [223].

In addition, the progression of CDDs might not occur at a consistent rate during a day. The administration of drugs with appropriate intervals on the basis of the circadian rhythm of CDDs is of prime interest in view of maximizing drug efficiency and reducing adverse side effects. Luchavova et al. [224] investigated the effect of the timing of teriparatide intake on the circadian rhythm of bone turnover in postmenopausal osteoporotic women. Their findings revealed that serum CTX indicated a circadian rhythm after evening teriparatide administration and a circasemidian rhythm after morning teriparatide administration, suggesting that the timing of teriparatide treatment may significantly change the rhythm of BTMs [224]. Therefore, more drugs should be considered for administration at an optimal time to provide maximum benefits for patients with CDDs. However, it is still unclear whether the circadian rhythm is changed under disease conditions. Hence, targeting the peak time of indicators of ARDs may be a better option.

### 5.2 Chrono‐Nanomedicine

The nanotechnology can function as a precision tool that enhances drug delivery by leveraging physiological rhythms. For example, the permeability of the blood‒brain barrier exhibits a circadian rhythm, which allows for the administration of nanocarriers during their most susceptible window, thereby increasing therapeutic efficacy for brain diseases such as AD [225]. To facilitate automated precision dosing, pulsatile nanodrug delivery systems have been developed. These systems are capable of releasing drugs in response to predetermined times or pathological signals, such as tumor recurrence, effectively translating chronopharmacology into clinically applicable strategies [226].

The frontier paradigm in this field regards nanomaterials as direct signaling sources capable of modulating the cellular clock. For example, the nanoplatform‐induced photothermal effect can induce Ca^2+^ influx, which directly interferes with and suppresses the expression of the core clock proteins Bmal1 and Clock. This disruption of the circadian clock in tumor cells creates optimal conditions for enhancing the efficacy of chemotherapy [227].

The deep integration of nanotechnology and chronobiology is pioneering a medical revolution, transitioning from merely conforming to biological rhythms to actively reshaping them. This integration not only offers unprecedented technological means to harness biological rhythms but also reveals entirely new possibilities for disease treatment by reprogramming the intrinsic temporal programs of cells. Consequently, this research opens vast frontiers for the future of personalized precision medicine.

### 5.3 Nonpharmacological Interventions

Behavioral interventions to sustain a circadian rhythm in the sleep‒wake cycle, shift work and feeding have been proposed as efficient approaches to prevent or treat several chronic diseases [228]. With respect to human health, good bedtime habits and sufficient sleep contribute to improving the circadian rhythm and stabilizing tissue deterioration. Sleep disruption and chronic intermittent hypoxia caused by obstructive sleep apnea (OSA) have been associated with human health [229, 230]. For example, OSA patients had significantly lower BMD, increased bone resorption and greater fracture risk than healthy individuals did [231, 232]. Thus, aggressive treatments for OSA may prove beneficial in addressing CDDs. Furthermore, as described above, night shift work and related circadian disruption are other potential risk factors for the development of CDDs [233, 234]. Given that night shift work is currently a common occupational phenomenon, it is important for employees with night shift work to pay close attention to their health. Daytime work seems to be a better choice than night shift work.

Moreover, the circadian rhythm of peripheral tissue is responsive to the feeding‒fasting cycle [235].Time‐restricted circadian feeding, which involves setting the feeding period to a defined interval without a reduction in caloric intake, is associated with systemic inflammation [236, 237]. For example, Adawi et al. [238, 239, 240] reported that intermittent fasting could help decrease psoriatic arthritis disease activity and hidradenitis severity. Since tissue homeostasis is closely related to systemic inflammation, the impact of intermittent fasting on tissue deterioration warrants further investigation [241]. In cardiovascular disease models, the protective role of intermittent fasting extends beyond mere caloric restriction. More importantly, it induces metabolic reprogramming in cardiomyocytes by modulating the AMPK and SIRT1 signalings. This process effectively alleviates ischemic injury, suppresses cardiac fibrosis, prevents adverse ventricular remodeling, and ultimately delays the progression of heart failure [242]. Time‐restricted feeding has been proven to be safe and may be a promising alternative behavioral intervention to regulate circadian rhythm and improve human health [228, 243]. Similarly, a ketogenic diet has been demonstrated to activate AMPK in a non‐small cell lung cancer model, which subsequently modulates the expression of the core circadian clock gene Bmal1. It functions as a tumor suppressor by activating intrinsic anticancer pathways through the regulation of apoptotic genes while also effectively restoring the circadian rhythm disrupted by tumor growth [244].

Whether through the adjustment of the scheduling of daily activities or dietary patterns, the core strategy lies in using regular external signals to calibrate and reinforce endogenous biological rhythms, thereby promoting health at the molecular, cellular, and systemic levels.

### 5.4 Translational Challenges and Future Directions

The relationship between circadian rhythm and tissue deterioration offers new insights into improving therapies for CDDs. However, several challenges remain regarding the clinical application of circadian rhythm‐based therapy. First, the influence of the circadian rhythm on health has been demonstrated across various species, including humans, rats, and mice, suggesting that the circadian rhythm may play a universal role in maintaining tissue homeostasis. However, most studies are still limited to animal experiments or small‐scale clinical trials, which lack long‐term follow‐up and robust data from larger samples. Further prospective cohort studies are essential to develop effective clinical therapies for CDDs that leverage circadian rhythm regulation. Besides, considerable variations among individuals exist in human biological rhythms, encompassing circadian rhythms and hormonal fluctuations, which are influenced by multiple factors, including genetics, age, environmental influences, and lifestyle choices. At present, the accurate anticipation and modeling of personalized rhythms are inadequately developed, complicating the establishment of a uniform treatment approach. Furthermore, efficient monitoring of biological rhythms requires the implementation of continuous and noninvasive methods. Although progress has been achieved through wearable technology, their reliability and accuracy still fall short. Finally, customized rhythm therapy may increase the costs associated with diagnosis and treatment, thereby posing challenges for implementation in areas with limited medical resources. Regarding these challenges, more efforts are needed to offer encouraging possibilities for the targeted and individualized use of biological rhythm therapy.

## 6 Conclusions and Future Perspectives

The circadian rhythm plays a crucial role in regulating tissue homeostasis and human health. Disruption of the circadian rhythm has been linked to CDDs. The influence of circadian rhythm on metabolic homeostasis, neuroendocrine signaling, immune and oxidative stress responses, tissue repair and autophagy activity is orchestrated by a group of clock genes, with Bmal1 serving as the central regulator.

However, several areas require further exploration. First, more molecular and cellular mechanisms through which the circadian rhythm regulates tissue deterioration need to be explored. In addition to the Bmal1/Clock–Per/Cry and Bmal1–REV‐ERB/ROR feedback loops mentioned above, a growing number of studies have identified many other clock genes, such as casein kinase 1 [245], albumin Dsite‐binding protein [246], and e4 promoter‐binding protein 4 [247], that are involved in the regulation of the circadian rhythm. However, their roles in tissue deterioration remain to be investigated. Recent evidence suggests that Bmal1 increases H2Bub1 levels by regulating the circadian‐controlled gene TTK, while H2Bub1 in turn positively modulates the expression of Bmal1 [248]. Thus, circadian genes and genes related to tissue deterioration can also form positive feedback loops to maintain tissue homeostasis. However, further studies are needed to fully determine how circadian rhythm impacts tissue deterioration. Second, tissue homeostasis depends on the balance of activities of different cell types, as well as their communication, which may also be affected by disruption of the circadian rhythm [249, 250]. The impact of the circadian rhythm on cell‒cell communication, such as MSC‒macrophage cross talk, and the potential mechanism also deserve exploration. Third, the regulation of endogenous antioxidant defensive systems represents an emerging area of research interest in the treatment of diseases [251, 252, 253]. Increasing evidence indicates that the circadian rhythm confers protection during aging through the modulation of antioxidant defense systems [254]. The vitagene network has been proposed to play an important role in defense mechanisms against oxidative stress and degenerative diseases during aging [255, 256]. Whether the circadian rhythm could impact tissue deterioration via the regulation of vitagen expression needs further investigation. Fourth, in addition to circadian rhythm, tissue metabolism is also reported to change with season [257, 258, 259]. From the perspective of biological rhythm, it is also necessary to pay more attention to the role of seasons and temporal changes in tissue deterioration. Fifth, fluctuations in temperature may also influence circadian rhythm and tissue metabolism. Increased expression of Per1/2 in the peripheral tissues of mice has been observed in those subjected to 41°C warm water [260], suggesting a potential impact of temperature on circadian rhythms. Further research is needed to assess the direct effects of temperature on circadian rhythms and human health.

Taken together, the current findings provide crucial insights into the influence of circadian rhythms on tissue homeostasis, presenting potential avenues for managing CDDs. Therefore, further advancements in research are essential to address circadian rhythm disruptions, aiming to prevent or delay the onset of CDDs.

## Author Contributions

KZ, BM, YX, and SW collected the data and wrote the manuscript. LL collected the data. SZ developed the figures. XL, HCM, and JH edited the manuscript and developed the figures. ACP and SK reviewed the manuscript. LC, GL, and SL conceived and supervised the study and revised the manuscript. All the authors read and approved the final manuscript.

## Ethics Statement

The authors have nothing to report.

## Conflicts of Interest

The authors declare no conflict of interest.

## Data Availability

The authors have nothing to report.
